# Familial novel androgen receptor gene variant associated with bilateral cryptorchidism and severe male infertility: A case report

**DOI:** 10.1016/j.eucr.2025.103235

**Published:** 2025-10-09

**Authors:** Zohor Azher

**Affiliations:** Medical Genetics Department, Faculty of Medicine, Umm Al-Qura University, Mecca, 24382, Saudi Arabia

**Keywords:** Androgen insensitivity, Partial androgen insensitivity syndrome, Androgen receptor gene, Cryptorchidism, Male infertility, WGS

## Abstract

Cryptorchidism is a common congenital anomaly linked to infertility and testicular cancer risk. Variants in the *androgen receptor* (AR) gene cause androgen insensitivity syndrome (AIS), ranging from complete (CAIS) to partial (PAIS) and mild (MAIS) forms. We report a male patient with infertility, severe oligoasthenoteratozoospermia, and bilateral cryptorchidism. Whole-genome sequencing revealed a novel AR missense variant (p.Tyr364His) in the N-terminal domain, predicted to cause partial receptor dysfunction. The same variant was found in his brother with cryptorchidism and PAIS features. This finding expands the AR mutational spectrum and emphasizes the need for early genetic evaluation and counseling in cryptorchidism.

## 1 Introduction

Cryptorchidism, or undescended testis, refers to the failure of one (unilateral) or both testes (bilateral) to descend into the scrotal position. It is among the most common congenital anomalies in male newborns, with a reported prevalence of 3.4–5.8 % in full-term infants and up to 30 % in premature neonates.^1^ However, spontaneous testicular descent occurs in approximately 80 % of affected infants during the first three months of life, thereby reducing the true incidence to around 1 % by that age.^2^

Untreated or late-treated cryptorchidism patients have significant long-term consequences, including a markedly increased risk of testicular germ cell tumors and impaired fertility. Spermatogenesis is often compromised in testes that remain undescended,^3^ and approximately 10 % of infertile men report a history of cryptorchidism and orchidopexy.^4^ The incidence of azoospermia in unilateral cryptorchidism is estimated at 13 %, whereas it may exceed 90 % in untreated bilateral cases,^5^ making cryptorchidism the most common etiological factor of spermatogenic impairment in adulthood. Given the high incidence of cryptorchidism and its serious complications, routine neonatal screening through genital exam and early treatment is strongly recommended for cryptorchid boys.

From an embryological perspective, testicular descent occurs in two distinct phases. The **transabdominal phase** (weeks 8–15 of gestation) is primarily driven by insulin-like factor 3 (INSL3), secreted by fetal Leydig cells, which promotes transabdominal migration of the gubernaculum (caudal genital ligament). At the same time, androgens contribute indirectly by inducing regression of the craniosuspensory ligament, thus facilitating testicular descent. The subsequent **inguinoscrotal phase** (weeks 25 to term) is predominantly androgen-dependent, aided by additional factors such as intra-abdominal pressure leading to anchoring of fetal testes into the scrotum.^6^ Disruption of these pathways, whether due to abnormal secretion or action of INSL3 or androgen hormones, results in failure of normal testicular migration within the scrotum.

Here, we report the clinical presentation and genetic findings of two brothers with severe oligoasthenoteratozoospermia and bilateral cryptorchidism. Whole-genome sequencing (WGS) revealed a novel missense variant in the androgen receptor (*AR*) gene, providing further insight into the molecular mechanisms underlying this condition.

### 1.1 Case description

A 30-year-old man presented with a two-year history of primary infertility, associated with anorgasmia and erectile dysfunction since marriage. His past medical history was remarkable for bilateral cryptorchidism, managed by right orchiectomy and left orchidopexy at the age of three years. He denied any history of testicular trauma, mumps orchitis, radiation, or chemotherapy, and reported normal pubertal development.

**Fig. 1:**
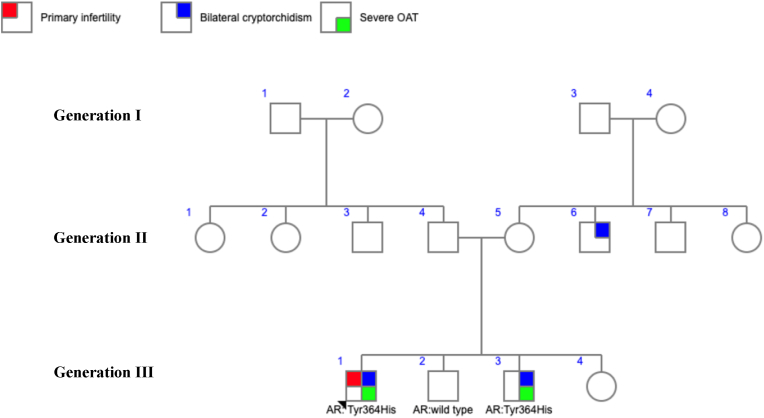
Pedigree of the family with the *AR* (c.1090T > C; p. Tyr364His) variant. Arrow: the proband. Severe OAT: Severe oligoasthenoteratozoospermia. AR: Androgen receptor gene.

Family history revealed non-consanguineous parents (Fig. 1). One brother (Individual III-2) had no history of cryptorchidism or infertility, while another brother (Individual III-3) had bilateral cryptorchidism corrected at age of three years; he is unmarried, thus his fertility status is undetermined.

Physical examination of the proband showed normal body hair distribution and phallus, with the left testis palpable within a tight scrotum. No clinical evidence of congenital vasal agenesis was observed. Semen analyses repeatedly demonstrated markedly reduced sperm count, poor motility, and abnormal morphology, consistent with severe oligoasthenoteratozoospermia (OAT) (Table 1). Hormonal evaluation showed elevated FSH and LH with normal testosterone levels (Table 1). Scrotal ultrasound showed the left testicle is small in size, measuring 2.7 x 2.5 × 1 cm. Cytogenetic analysis revealed a male karyotype (46,XY) with no chromosomal abnormalities, and Y-chromosome microdeletion testing was negative. Two assisted reproductive technology (ART) attempts with intracytoplasmic sperm injection (ICSI) using ejaculated sperm were unsuccessful.

**Table 1:** Semen analysis and hormone profile of a) the proband (Individual III-1) and b) his brother (Individual III-3). Semen analysis is performed on two separate occasions at least two months apart. FSH, follicle-stimulating hormone; LH, luteinizing hormone; T, total testosterone; PRL, prolactin.

(a) Proband (Individual III-1):				
Semen Parameters	1st Results	2nd Results	Hormone profile	Results
Volume (ml)	0.5	2	FSH (mIU/ml)	43.40
Sperm concentration (per ml)	700,000	500,000	LH (mIU/ml)	14.84
Total motility (%)	10 %	11 motile sperm/slide	T (nmol/ml)	20.99
Normal morphology (%)	1 %	0 %	PRL (ng/ml)	9.91

(b) Brother (Individual III-3):				
Semen Parameters	1st Results	2nd Results	Hormone profile	Results
Volume (ml)	1	1	FSH (mIU/ml)	3.50
Sperm concentration (per ml)	800,000	4,000,000	LH (mIU/ml)	5.51
Total motility (%)	40 %	40 %	T (nmol/ml)	18.20
Normal morphology (%)	1 %	1 %	PRL (ng/ml)	16.16

WGS was subsequently performed on DNA extracted from a buccal swab of the proband. A hemizygous missense variant in the *AR* gene (NM_000044.6: c.1090T > C; p.Tyr364His) was identified, predicted to substitute tyrosine with histidine at codon 364. This variant is rare, with a very low minor allele frequency (MAF) of 0.0000123 in gnomAD. Multiple in silico tools predicted a deleterious effect (Table 2).

**Table 2:** In silico prediction tools and population databases of familial AR variant (p. Tyr364His). ACMG: American College of Medical Genetics guidelines.

Gene/Sequence ID	AR/NM_000044.6
DNA change	c.1090T > C
Protein change	p. Tyr364His
SIFT	Damaging
PolyPhen-2	Probably damaging
CADD(PHRED)	25.3
REVEL	0.67
GenomAD	0.0000123
ACMG criteria	PM2, PP1, PP3, PP4

Segregation analysis revealed the *AR* variant in Individual III-3, while it was absent in Individual III-2. The maternal sample was unavailable for testing. A subsequent clinical evaluation of Individual III-3 revealed a normal general examination and phallus, with testicular volumes of 8 cc (right) and 18 cc (left). His semen analysis revealed abnormal parameters consistent with severe OAT. Hormonal evaluation was within normal limits, except for a mild elevation in prolactin (Table 1).

## 2 Discussion

*AR* gene, located on the long arm of the X chromosome (Xq12), encodes the androgen receptor protein, which is a member of the nuclear steroid receptor superfamily that functions as a transcriptional regulatory factor.^7^^,^^8^ Upon binding to steroid hormones (androgens) such as testosterone or dihydrotestosterone, the receptor undergoes activation and forms a hormone–receptor complex. This complex then dissociates from accessory proteins, translocates into the nucleus, where it regulates the transcription of androgen-responsive genes.^9^ These genes play a crucial role in male sex differentiation during embryogenesis (masculinization), the development of secondary sexual characteristics at puberty (virilization), and spermatogenesis.

**Androgen insensitivity syndrome (AIS)** is the most common disorder of sexual development (DSD) in individuals with a 46, XY karyotype.^10^^,^^11^ It is an X-linked recessive condition caused by pathogenic variants in the *AR* gene, which result in androgen receptor dysfunction and, consequently, resistance to androgen action. Based on clinical heterogeneity, AIS is classified into three phenotypes: complete androgen insensitivity syndrome (CAIS), partial androgen insensitivity syndrome (PAIS), and mild androgen insensitivity syndrome (MAIS). CAIS occurs in approximately 2–5 per 100,000 genetic males, while PAIS is thought to be at least as common. In contrast, MAIS is much less frequently reported.^12^ Individuals with CAIS typically present with normal female external genitalia with the absence of internal female genital structures and intra-abdominal, inguinal, or labial testes.^13^ PAIS is the incomplete form of androgen resistance and represents 10 % of individuals with AIS,^14^ which results from the partial inability of body cells to respond to androgens. The PAIS clinical manifestations vary according to the degree of AR residual function. Phenotypes range from predominantly female with evidence of external genital masculinization (such as clitoromegaly or posterior labial fusion) to predominantly male with genital anomalies including hypospadias, cryptorchidism, or micropenis, often associated with gynecomastia and impaired spermatogenesis. Patients with MAIS usually exhibit typical male external genitalia but may develop gynecomastia and infertility in adulthood.^13^ In the present report, the two brothers exhibited bilateral cryptorchidism and severe OAT, consistent with a **partial phenotype of AIS.**

The reproductive hormone profile in CAIS and PAIS patients is similar.^15^^,^^16^ It is characterized by elevated or normal serum testosterone levels associated with normal or high serum Luteinizing hormone (LH) levels reflecting androgen receptor dysfunction with normal androgen secretion.^17^ In AIS patients, follicle-stimulating hormone (FSH) and estradiol levels tend to be normal or slightly elevated for males.^11^ The proband in the present family demonstrated elevated FSH and LH levels with normal serum testosterone, a pattern consistent with both androgen resistance and impaired spermatogenesis. In contrast, the second affected sibling (III-3) showed a normal hormonal profile, which has also been reported in other AIS cases.

The *AR* gene *s*pans approximately 90 kb of genomic DNA and comprises eight exons. It encodes the AR protein, a large polypeptide of 920 amino acids organized into four functional domains: the N-terminal transactivation domain (NTD), the DNA-binding domain (DBD), the hinge region (HR), and the ligand-binding domain (LBD). More than 1000 AR variants have been submitted in the AR database (ARDB; http://www.mcgill.ca/androgendb, updated September 2014), with approximately half reported in association with AIS and the remainder linked to other AR-related disorders, including spinal and bulbar muscular atrophy (Kennedy's disease), breast cancer, and premature ovarian failure^18^

*AR* variants are distributed across all exonic regions and affect each of the protein domains. The majority, however, are localized within the NTD (encoded by exon 1) and the LBD (encoded by exons 4–8). The NTD alone constitutes nearly half of the receptor and plays a critical role in regulating transcription of androgen-responsive genes. Consequently, structural or functional alterations in this domain significantly disrupt the androgen signaling pathway and often result in severe AR dysfunction. Indeed, most mutations in (exon1) have been associated with the CAIS phenotype. Nevertheless, distinct variants within the same coding region may also present with partial or mild forms of AIS.^18^ Frameshift and nonsense variants in the NTD introduce premature stop codons, leading to truncated, non-functional proteins and complete androgen receptor inactivation, thereby causing the CAIS phenotype.^19^ In contrast, missense variants in this domain may lead to either complete or partial androgen resistance, manifesting clinically as CAIS or PAIS, respectively.^20^ In the present family, we identified a novel missense variant, **p.Tyr364His**, within the NTD domain of the AR protein. This variant has not been previously reported and is predicted to partially impair AR function, thereby contributing to the PAIS phenotype. Furthermore, this variant expands the mutational spectrum of *AR* and further supports the prior evidence that missense variants in NTD can lead to an incomplete form of androgen resistance.

## 3 Conclusion

This study reports a novel familial variant in the AR gene, underscoring the importance of genetic evaluation in newborns presenting with cryptorchidism, with or without associated systemic manifestations. Early molecular diagnosis not only enables accurate prognosis but also facilitates the identification of related complications such as infertility. Furthermore, we emphasize the value of genetic screening in at-risk family members to allow for early diagnosis, appropriate counseling, and timely clinical management.

## Author contributions

The author (corresponding author) was solely responsible for the conception and design of the study, collecting and interpreting clinical and genetic data, writing the manuscript draft, reviewing and editing the final version.

## Funding sources

This research did not receive any specific grant from funding agencies in the public, commercial, or not-for-profit sectors.

## Glossary:

ACMG: American College of Medical Genetics guidelines
AIS: Androgen insensitivity syndrome
AR: Androgen receptor
ART: Assisted reproductive technology
CAIS: Complete androgen insensitivity syndrome
DBD: DNA-binding domain
DSD: Disorder of sexual development
FSH: Follicle-stimulating hormone
HR: Hinge region
ICSI: Intracytoplasmic sperm injection
INSL3: Insulin-like factor 3
LBD: Ligand-binding domain
LH: Luteinizing hormone
MAF: Minor allele frequency
MAIS: Mild androgen insensitivity syndrome
NTD: N-terminal transactivation domain
OAT: Oligoasthenoteratozoospermia
PAIS: Partial androgen insensitivity syndrome
WGS: Whole-genome sequencing
